# Prescriptions of psychopharmacologic drugs in Austria in 2019 and 2020 – Implications of the COVID-19 pandemic

**DOI:** 10.1192/j.eurpsy.2022.2328

**Published:** 2022-10-25

**Authors:** Dietmar Winkler, Berthold Reichardt, Max Rothenberg, Dan Rujescu, Edda Pjrek

**Affiliations:** 1 Department of Psychiatry and Psychotherapy, Medical University of Vienna, Vienna, Austria; 2 Austrian Social Health Insurance Fund (Österreichische Gesundheitskasse), Eisenstadt, Austria

**Keywords:** Austria, COVID-19, lockdown, prescriptions

## Abstract

**Background:**

Measures to reduce the spread of the SARS-CoV-2 virus have an impact on the mental health of the general population. Drug prescription rates can be used as a surrogate marker to estimate help seeking and health parameters of a population. The aim of this study was to compare psychopharmacologic drug prescriptions in Austria from the start of the pandemic in 2020 over time and with the previous year and to investigate the impact of the COVID-19 lockdowns in 2020.

**Methods:**

Data from the three largest public health insurances in Austria, covering over 98% of the general population, were analyzed. A total of 1,365,294 patients with a prescription of a psychopharmacologic drug in the months March to December in 2019 and 2020 were selected.

**Results:**

There was no significant change in prescribed defined daily doses (DDDs) during the lockdowns. However, there was a stockpiling effect before and at the beginning of lockdown 1. The number of new patients initiating psychopharmacologic treatment was significantly reduced during lockdown 1 but not during lockdown 2.

**Conclusions:**

The first COVID-19 lockdown in 2020 functioned as a barrier for new psychiatric patients seeking help, whereas the patients with ongoing treatments did not have significant problems. These results have to be taken into account for future planning, but follow-up studies are needed, as our results could be indicative of a change in the effect of the protective measures on the utilization of the healthcare system over time.

## Introduction

The SARS-CoV-2 virus has spread worldwide since December 2019. The first COVID-19-positive patients were diagnosed in Austria on February 25, 2020. Since then, the COVID-19 pandemic has been a concern for the Austrian health system as part of the global pandemic. Due to high pressure on healthcare providers and on critical infrastructure including an imminent shortage of hospital and intensive care unit beds, the Austrian government decided on two lockdowns in 2020.

Threats to public health such as the COVID-19 pandemic can significantly affect the feeling of security, well-being, and mental health of the general population: Traunmüller et al. [1] performed an online survey of the Austrian population and found that 37.7% of the participants reported a severe psychological impact of the COVID-19 pandemic and 10% were considered to suffer from depression, anxiety, or stress. Pieh et al. showed a marked increase in depressive and anxiety symptoms as well as clinical insomnia during the first COVID-19 lockdowns in Austria [2] and in the UK [3] compared to a reference period before. These detrimental health consequences seemed to persist even months after the end of the lockdown measures [4]. Moreover, two studies showed that stress and loneliness as a result of social restrictions in Austria were predictive of future depressive symptoms [5, 6].

The emotional stress factors that resulted from the pandemic included not only the fear of infection with the new virus and the associated fear of possible negative health consequences up to one’s own death or that of loved ones, but also considerable uncertainty due to the restriction of personal freedom by protective measures and the sometimes contradicting messages from authorities and politics. Furthermore, there were growing concerns about the financial and economic sequelae of the pandemic as the number of unemployment increased during the second quarter of 2020 in Austria [7]. It is conceivable that these stress factors could have had an even greater effect on predisposed individuals with pre-existing mental illnesses [8].

Mental healthcare providers play an important role in managing these emotional stressors. However, the World Health Organization (WHO) has pointed out that COVID-19 has been disrupting mental health services in most countries [9]. In Austria, the burden on the health system was significantly increased at the beginning of the spread of SARS-CoV-2 due to the shortage of medical protective equipment, which led to a reduction of services of hospitals and outpatient clinics [10]. This and the barrier effect of the restrictions on freedom of movement of people themselves during the lockdowns might have led to an undertreatment of people with mental illnesses.

The effects of the COVID-19 pandemic on the utilization of the health system in connection with mental illnesses and prescriptions of psychopharmacologic drugs have not been adequately investigated before. The aim of the present study was to analyze prescriptions of psychopharmacologic drugs in Austria during the year 2020, the first year of the COVID-19 pandemic, in comparison to the previous year 2019 and to draw inferences from these prescription rates to the utilization of the mental health system.

## Method

This study was approved by the Ethics Committee of the Medical University of Vienna (EC No. 2153/2020). A nationwide analysis of anonymized prescription data of all patients insured by the three large public health insurances in Austria, that is, Österreichische Gesundheitskasse (ÖGK), Versicherungsanstalt öffentlich Bediensteter, Eisenbahnen und Bergbau (BVAEB), and Sozialversicherung der Selbständigen (SVS), in 2019 and 2020 was performed.

8,773,427 persons (98.8% of the inhabitants of Austria) were insured by one of the three aforementioned health insurances in 2019 and 8,780,142 persons (98.5%) in 2020. Patients with a prescription of a psychopharmacologic drug, that is, drugs from the following ATC subgroups were selected: N06A (antidepressants), N05A (antipsychotics), N05B/C (anxiolytics, hypnotics), N06B (psychostimulants), N06D (antidementia drugs), and N07B (drugs against substance addiction). For this open cohort, data from each prescription for the study period were retrieved. Defined daily doses (DDDs) were derived from each prescription and summed up according to the recommendations of the World Health Organization [11].

Statistical analyses were performed using the R Project for Statistical Computing (version 4.2.1) [12] together with the packages lubridate [13], pheatmap [14], reshape2 [15], and ggplot2 [16]. Descriptive statistics were calculated and we compared March to December of 2020 (“2020/03–12”) with the same months of 2019 (“2019/03–12”) with non-parametrical univariate statistical tests (chi-square test, Mann–Whitney *U* test or Wilcoxon signed rank test). The level of significance (two-tailed) was set to *p* ≤ 0.05.

The two lockdowns in Austria in the year 2020 (lockdown 1 from March 16, 2020 to May 01, 2020, calendar weeks 12 to 18; lockdown 2 from November 17, 2020 to December 06, 2020, weeks 47 to 49) were defined as periods of interest. To evaluate time-dependent changes in 2020 in prescribed DDDs and number of new patients as hypothesized *a priori*, we calculated two generalized linear models employing quasi-Poisson regression. Weekly data of DDDs and new patients, respectively, were taken as dependent variables, while calendar week with sequential numbering of the weeks and an indicator variable for the periods of interest functioned as covariates. The indicator included the two COVID-19 lockdowns and 2 weeks before and after the lockdowns as pre- and post-lockdown periods with the non-lockdown weeks as reference. The levels of this indicator variable were tested for statistical significance. Diagnostics of residuals were performed and derived models were checked for lack of autocorrelation. Results are presented as counts, percentages, or arithmetic mean ± standard deviation where appropriate.

## Results

A total of 1,120,535 patients (12.8% of all insured persons) received at least one prescription of a psychopharmacologic drug in 2019/03–12 and 1,085,675 patients (12.4%, −3.1%; χ^2^_1_ = 816.33, *p* < 0.0001) in 2020/03–12 (Table 1 shows respective figures for the whole years 2019 and 2020 for comparison). The percentage of patients with a prescription aged 18 and above was 15.1% in 2019/03–12 and 14.6% in 2020/03–12. The total number of study subjects was 1,365,294.

**Table 1.** Prescriptions of psychopharmacologic drugs in Austria in 2019 and 2020.

	2019		2020		Change
Austrian general population [28]	8,877,637		8,916,845		+0.44%
Persons with health insurance	8,773,427	98.8%^a^	8,780,142	98.5%	+0.08%
Females	4,451,188	50.7%	4,453,980	50.7%	+0.06%
Males	4,322,239	49.3%	4,326,162	49.3%	+0.09%
Patients with prescriptions of psychotropic drugs	1,176,146	13.4%^b^	1,139,319	13.0%	−3.1%
Females	735,779	62.6%	713,759	62.6%	−3.0%
Males	440,361	37.4%	425,556	37.4%	−3.4%
Age (years, μ ± SD)	59.8 ± 20.5		60.5 ± 20.1		

a Percent of the general population.

b Percent of all insured persons.

9,506,129 prescriptions were issued in 2019/03–12 and 9,109,735 in 2020/03–12 (−4.2%; *V* = 737, *p* = 0.004). Prescriptions of ATC subgroups of psychopharmacologic drugs and data by gender are displayed in Table 2. Time course of prescriptions of ATC subgroups in 2020 is presented in Supplementary Figure 1. Here, a decrease in the prescriptions of anxiolytics and hypnotics of 12.0% (*V* = 927, *p* < 0.0001) and a decrease in antidementia drugs of 6.9% (*V* = 774, *p* = 0.0008) were most notable. The prescriptions contained 291,722,002 DDDs in 2019/03–12 and 286,450,149 in 2020/03–12 (−1.8%; *V* = 638, *p* = 0.097); further data for subgroups in Table 3).

**Table 2.** Number of prescriptions of psychopharmacologic drugs classes in Austria in the months March to December in 2019 and 2020.

	Total			Females			Males		
	2019/03–12	2020/03–12		2019/03–12	2020/03–12		2019/03–12	2020/03–12	
Total	9,506,129	9,109,735	−4.2%	6,013,274	5,767,045	−4.1%	3,492,835	3,342,673	−4.3%
Antidepressants (N06A)	5,065,046	4,933,478	−2.6%	3,378,813	3,297,342	−2.4%	1,686,222	1,636,119	−3.0%
Antipsychotics (N05A)	1,604,315	1,588,852	−1.0%	901,539	891,378	−1.1%	702,776	697,474	−0.8%
Anxiolytics (N05B) and hypnotics (N05C)	1,375,187	1,210,616	−12.0%	849,816	749,293	−11.8%	525,371	461,323	−12.2%
Antidementia drugs (N06D)	1,131,344	1,053,812	−6.9%	758,211	707,495	−6.7%	373,132	346,317	−7.2%
Others (N06B and N07B)	330,237	322,977	−2.2%	124,895	121,537	−2.7%	205,334	201,440	−1.9%

**Table 3.** Defined daily doses (DDDs) of prescribed psychopharmacologic drugs in Austria in the months March to December in 2019 and 2020.

	Total			Females			Males		
	2019/03–12	2020/03–12		2019/03–12	2020/03–12		2019/03–12	2020/03–12	
Total	291,722,002	286,450,149	−1.8%	182,832,460	179,872,718	−1.6%	108,888,844	106,576,663	−2.1%
Antidepressants (N06A)	178,777,672	177,707,840	−0.6%	118,669,761	118,279,653	−0.3%	60,107,583	59,427,419	−1.1%
Antipsychotics (N05A)	31,624,152	31,896,314	0.9%	16,111,898	16,249,260	0.9%	15,512,254	15,647,054	0.9%
Anxiolytics (N05B) and hypnotics (N05C)	32,721,180	29,532,437	−9.7%	18,500,862	16,616,733	−10.2%	14,220,318	12,915,704	−9.2%
Antidementia drugs (N06D)	42,523,158	41,094,775	−3.4%	27,969,087	27,098,848	−3.1%	14,553,991	13,995,927	−3.8%
Others (N06B and N07B)	6,075,840	6,218,783	2.4%	1,580,852	1,628,224	3.0%	4,494,698	4,590,559	2.1%

**Figure 1.**
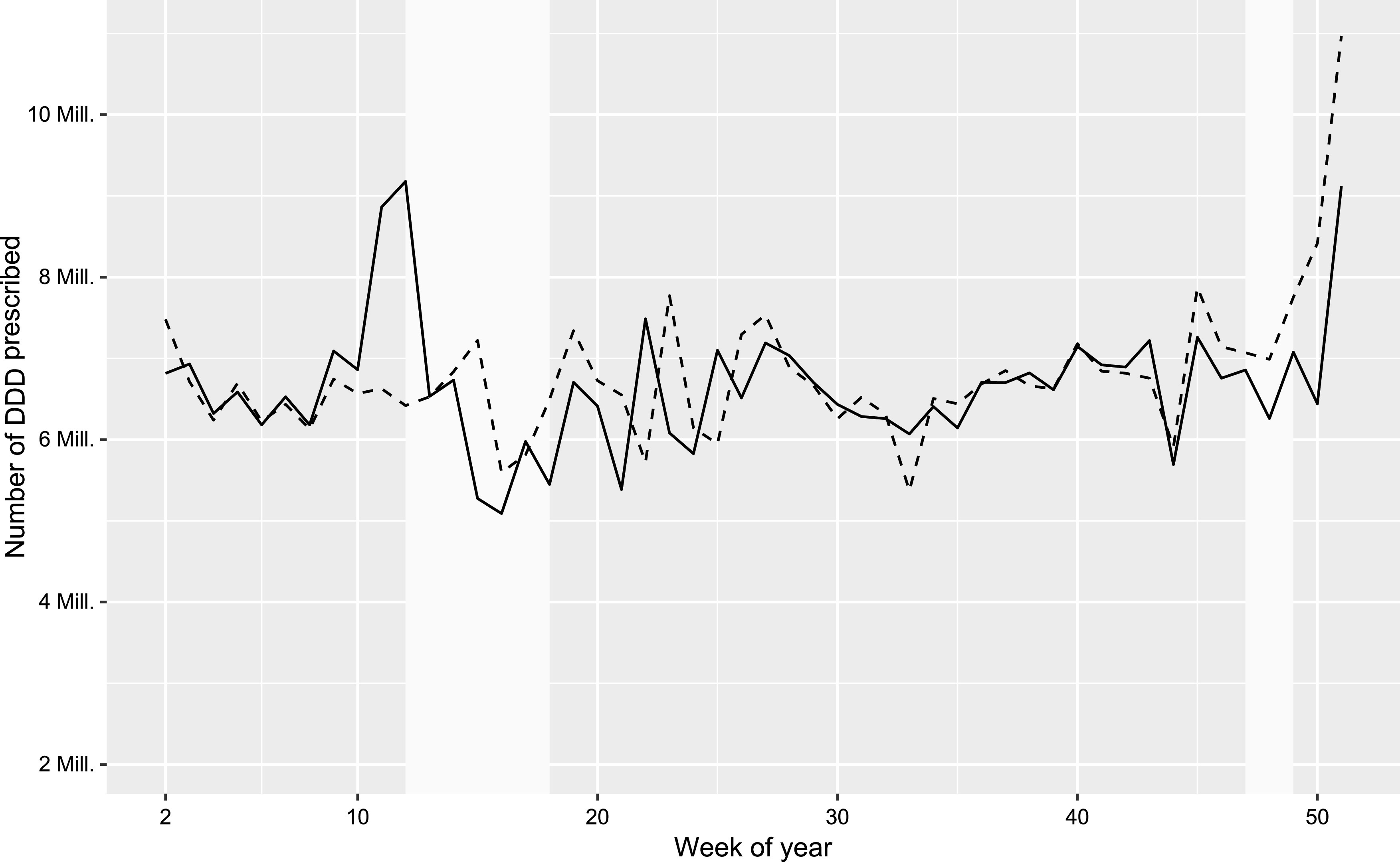
Number of defined daily doses (DDDs) of prescribed psychopharmacologic drugs in Austria in 2019 (dashed line) and 2020 (solid line) by week of year. The time periods of lockdown 1 (March 16, 2020 to May 01, 2020, weeks 12–18) and lockdown 2 (November 17, 2020 to December 06, 2020, weeks 47–49) are shaded light-gray.

Prescribed DDDs did not change significantly during lockdown 1 (*B* = 0.008, *t* = 0.115, *p* = 0.909) or lockdown 2 (*B* < −0.001, *t* = −0.004, *p* = 0.997) but showed an increase of +20.8% in the 2 weeks before lockdown 1 (*B* = 0.236, *t* = 2.204, *p* = 0.033; DDDs in weeks 10 and 11 in 2020: 7,861,490 ± 1,414,380, DDDs of all other weeks in 2020: 6,508,664 ± 983,772.6; Figure 1).

**Figure 2.**
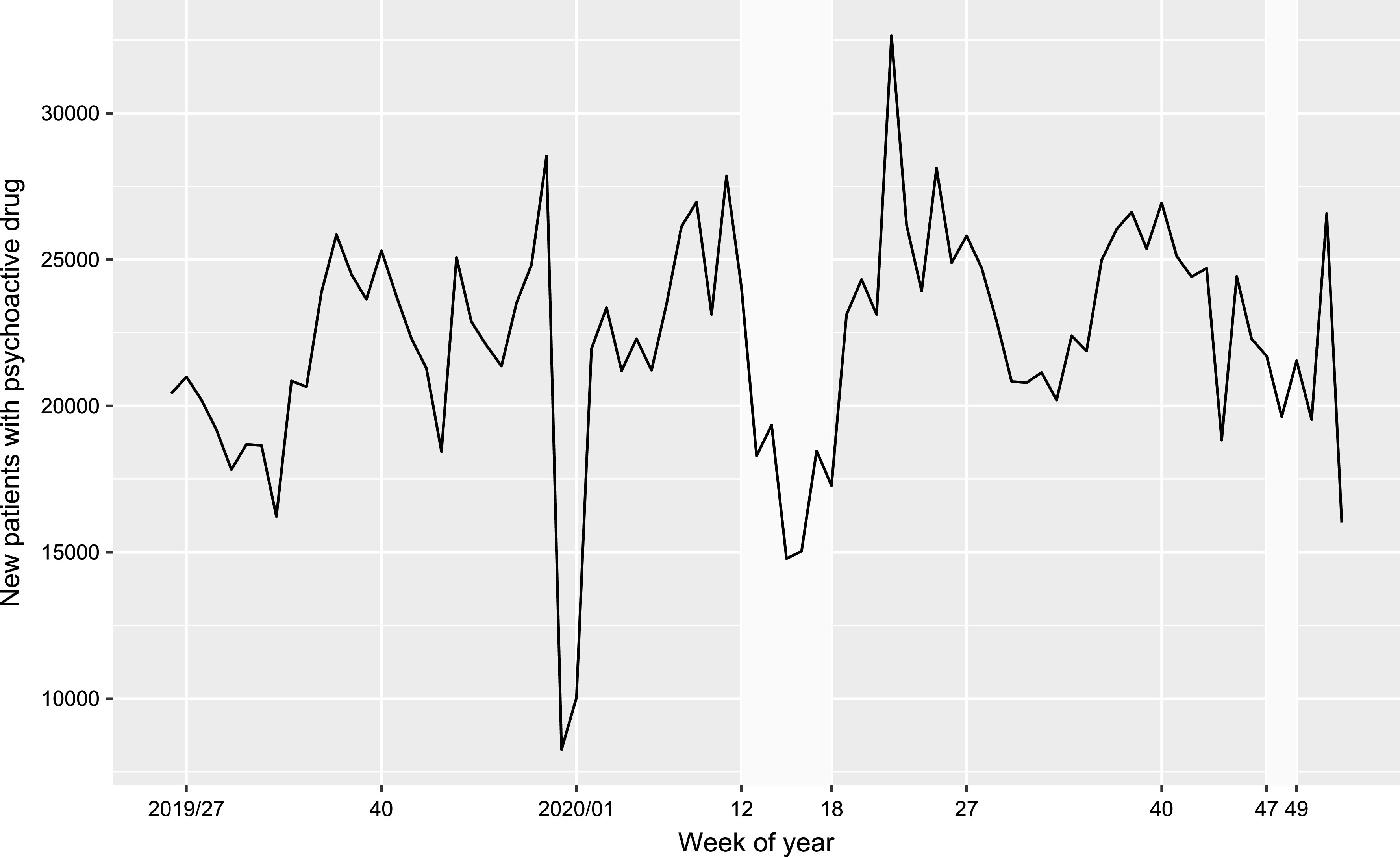
New patients (i.e., with no prescription within the last 60 days) receiving a prescription of a psychotropic drug during the second half of 2019 and 2020 by week of year. Lockdown 1 (weeks 12–18) and lockdown 2 (weeks 47–49) are shaded light-gray.

We defined new patients as patients with no prescription for a psychotropic drug during the last 60 days (Figure 2). There was a significant reduction in new patients by −22.3% compared to the non-lockdown weeks during lockdown 1 (*B* = −0.240, *t* = −3.163, *p* = 0.003) but no significant reduction (−10.4%) during lockdown 2 (*B* = −0.134, *t* = −1.233, *p* = 0.224).

**Figure 3.**
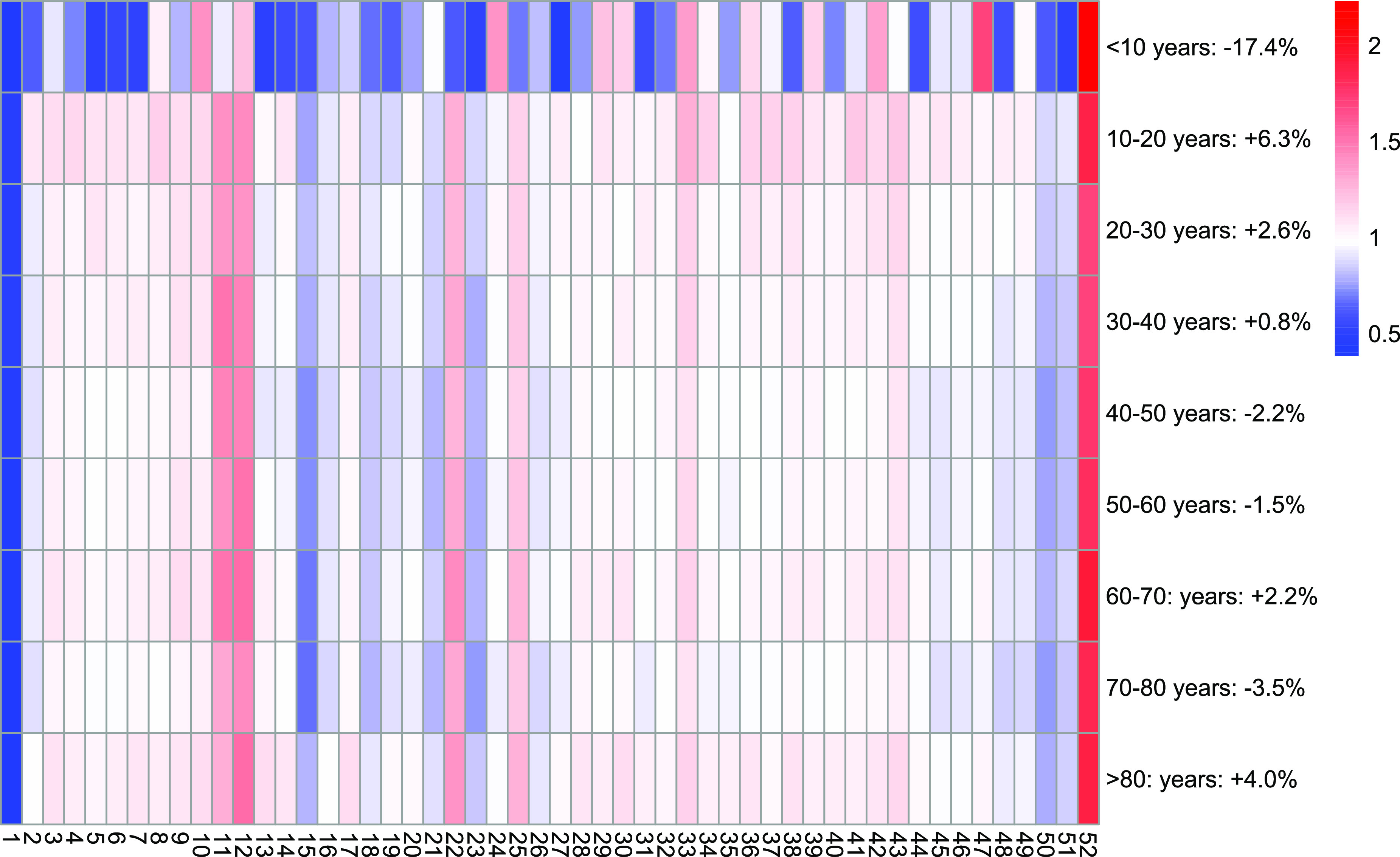
Heat map of ratio in prescribed DDDs of antidepressants in the year 2020 versus 2019 by age groups and weeks of the year. The legend also shows the percental change of the prescribed DDDs from 2019/03–12 to 2020/03–12 in the age groups. Lockdown weeks in 2020 were 12–18 and 47–49.

As antidepressants constitute a major part of all prescribed psychopharmacologic drugs and prescriptions of this drug class are likely to be influenced by stress and fear regarding the pandemic, we performed an exploratory analysis of prescribed DDDs of antidepressants by week and age class (Figure 3): We saw a decrease of −17.4% in children <10 years old from 2019/03–12 to 2020/03–12 (weekly DDDs of antidepressants 654.3 ± 157.6; *V* = 725, *p* = 0.002) and an increase of +6.3% in adolescents (10–20 years, weekly DDDs 55,964.4 ± 7,692.0; *V* = 264, *p* = 0.011). Furthermore, changes in the age groups of the adult study population were smaller.

## Discussion

To our best knowledge, this is the first study to investigate the effect of COVID-19 lockdowns on a national sample of psychopharmacologic prescription data. However, attempts to make assumptions on illnesses and to estimate health parameters by analyzing prescription rates of psychopharmacologic drugs [17–20] and studies on the effect of COVID-19 restrictions on the utilization of the health system [21] have been done before.

During the lockdowns in Austria everybody was required to stay at home except for averting an immediate danger to life and property, caring for and providing assistance to people in need of support as well as exercising family rights and fulfilling family obligations, covering the necessary basic needs of daily life (e.g., buying food or medication, going to a medical doctor), professional purposes, and staying outdoors for physical and mental relaxation (only individually or with people of one’s own household) [22].

In 2020/03–12, 3.1% fewer patients received a prescription compared to the same period in the previous year. We observed a reduction in prescriptions (−4.2%) during the pandemic in 2020, but this was partly offset by the prescribed DDDs. The time course of prescribed DDDs in 2020 showed a stockpiling effect regarding medication (across all drug subgroups) before lockdown 1. However, this increase did not end at the beginning of lockdown 1, but showed a peak in the first week of lockdown 1 (week 12) with 9,178,676 prescribed DDDs (+42.3% compared to the rest of 2020/03–12). This effect was not noticeable again before lockdown 2. This can be interpreted as an emotional response to an unknown situation similar to the shopping sprees that have been reported in connection with the COVID-19 lockdowns [23, 24]. It has to be noted that Austria has not seen any curfew-like measures for over 75 years since the end of World War II. The increase in prescriptions at the end of the year (Figure 1) is due to many people, who reach the prescription fee cap towards the end of the year and want to take advantage of this.

Our analysis of new patients entering treatment suggests that the pandemic and the first lockdown have particularly been a major barrier for this patient population. We see a significant decrease in new patients during lockdown 1 but this effect was smaller and statistically not significant during the second lockdown. It is not clear, if this is due to lockdown 2 being shorter or if the barrier effect of the lockdowns was fading over time. This has to be investigated by follow-up studies. Furthermore, a reduction in new patients at the turn of the year 2019/2020 can be discerned from Figure 2, but this can be explained by the numerous public holidays in this period.

Our finding of a reduction in antidepressant prescriptions among children (<10 years old) must be viewed against the background of the very small prescribing numbers in this age group. It is doubtful whether the slight increase in the use of antidepressants among adolescents (10–20 years old) corresponds to the recently reported increased psychological stress on adolescents in the context of the pandemic [25–27], since there was already an increase in the prescription of antidepressants in this age group before lockdown 1 (Figure 3).

The number of prescriptions and the prescribed DDDs of anxiolytics and hypnotics fell by more than 10% in 2020 compared to 2019. However, this reduction cannot be reliably attributed to a change in prescription habits: Our data includes only prescription drugs that are paid for by health insurance companies. In 2020, the price of two major anxiolytics, namely Psychopax drops and Praxiten 15 mg tablets, fell below the deductible limit, which is why these two drugs are no longer included in our data. Furthermore, there were supply problems with another important anxiolytic, namely Praxiten 50 mg tablets around the middle of 2020, which may also have significantly influenced the prescription data.

A strength of this analysis is the very large, nationwide sample, covering more than 98% of the Austrian general population. However, our study is limited in several ways: Despite their psychiatric usage, we did not include prescriptions for anticonvulsant drugs, because we could not have reliably differentiated an application as a neurological treatment. Over-the-counter medication and prescription drugs below the price of the deductible limit are not included in our data. The changes during the pandemic are very likely the result of several overlapping effects, such as altered help-seeking and potential barrier effects. Still, our study can only display the sum of these effects without the possibility for further differentiation.

Our results indicate that the pandemic and in particular lockdowns can impair mental health of the general population as they might pose a barrier to consulting health professionals for patients without prior treatment.

## Data Availability

Individual patient-level data are not available due to patient confidentiality and data protection agreements in place.

## Supplementary material

For supplementary material accompanying this paper visit https://doi.org/10.1192/j.eurpsy.2022.2328. click here to view supplementary material

## References

[1] Traunmüller C, Stefitz R, Gaisbachgrabner K, Schwerdtfeger A. Psychological correlates of COVID-19 pandemic in the Austrian population. BMC Public Health. 2020;20:1395. 32928180 10.1186/s12889-020-09489-5 PMC7487438

[2] Pieh C, Budimir S, Probst T. The effect of age, gender, income, work, and physical activity on mental health during coronavirus disease (COVID-19) lockdown in Austria. J Psychosom Res. 2020;136:110186. 32682159 10.1016/j.jpsychores.2020.110186 PMC7832650

[3] Pieh C, Budimir S, Delgadillo J, Barkham M, Fontaine JRJ, Probst T. Mental health during COVID-19 lockdown in the United Kingdom. Psychosom Med. 2021;83:328–37. 33009276 10.1097/PSY.0000000000000871

[4] Pieh C, Budimir S, Humer E, Probst T. Comparing mental health during the COVID-19 lockdown and 6 months after the lockdown in Austria: a longitudinal study. Front Psychiatry. 2021;12:625973. 33859579 10.3389/fpsyt.2021.625973 PMC8042148

[5] Probst T, Budimir S, Pieh C. Depression in and after COVID-19 lockdown in Austria and the role of stress and loneliness in lockdown: a longitudinal study. J Affect Disord. 2020;277:962–3. 33065839 10.1016/j.jad.2020.09.047 PMC7487145

[6] Mayerl H, Stolz E, Freidl W. Longitudinal effects of COVID-19-related loneliness on symptoms of mental distress among older adults in Austria. Public Health. 2021;200:56–8. 34678551 10.1016/j.puhe.2021.09.009 PMC8479381

[7] Arbeitsmarktservice Österreich (AMS Österreich). Arbeitsmarktdaten. 2020.

[8] Torales J, O’Higgins M, Castaldelli-Maia JM, Ventriglio A. The outbreak of COVID-19 coronavirus and its impact on global mental health. Int J Soc Psychiatry. 2020;66:317–20. 32233719 10.1177/0020764020915212

[9] World Health Organization. COVID-19 disrupting mental health services in most countries, WHO survey; 2020.

[10] Eglau K. Erste Analyse der Auswirkungen des Lockdowns während der COVID-19-Pandemie auf die stationäre Spitalsversorgung anhand ausgewählter Bereiche. Vienna: Gesundheit Österreich; 2020.

[11] WHO Collaborating Centre for Drug Statistics Methodology. ATC/DDD index 2022. https://www.whocc.no/.

[12] R Core Team. R: a language and environment for statistical computing. Vienna, Austria: R Foundation for Statistical Computing; 2022. https://www.R-project.org/.

[13] Garrett G, Hadley W. Dates and times made easy with lubridate. J Stat Softw. 2011;40:1–25.

[14] Raivo K. pheatmap: pretty heatmaps. R package version 1.0.12. 2019, https://CRAN.R-project.org/package=pheatmap.

[15] Wickham H. Reshaping data with the reshape package. J Stat Softw 2007;21:1–20.

[16] Wickham H. ggplot2: elegant graphics for data analysis. New York: Springer; 2009. https://ggplot2.tidyverse.org/.

[17] Conn DK, Ferguson I, Mandelman K, Ward C. Psychotropic drug utilization in long-term-care facilities for the elderly in Ontario, Canada. Int Psychogeriatr. 1999;11:223–33. 10547123 10.1017/s1041610299005797

[18] Han KM, Kim KH, Lee M, Lee SM, Ko YH, Paik JW. Increase in the prescription rate of antidepressants after the Sewol Ferry disaster in Ansan, South Korea. J Affect Disord. 2017;219:31–6. 28505500 10.1016/j.jad.2017.05.026 PMC7112638

[19] van den Driest JJ, Schiphof D, de Wilde M, Bindels PJE, van der Lei J, Bierma-Zeinstra SMA. Antidepressant and anticonvulsant prescription rates in patients with osteoarthritis: a population-based cohort study. Rheumatology (Oxford). 2021;60:2206–16. 33175150 10.1093/rheumatology/keaa544 PMC8121444

[20] Winkler D, Reichardt B, Kranz GS, Bartova L, Kasper S, Pjrek E. Seasonality of antidepressant prescriptions and sick leaves. J Psychiatr Res. 2019;111:128–33. 30738345 10.1016/j.jpsychires.2019.01.020

[21] Chow MW, Noorthoorn EO, Wierdsma AI, van der Horst M, de Boer N, Guloksuz S, et al. Impact of the first COVID-19 outbreak on mental health service utilisation at a Dutch mental health centre: retrospective observational study. BJPsych Open. 2021;7:e213. 34784994 10.1192/bjo.2021.1049 PMC8632375

[22] Bundesgesetz betreffend vorläufige Maßnahmen zur Verhinderung der Verbreitung von COVID-19 (COVID-19-Maßnahmengesetz, COVID-19-MG). Paragraph 6, Ausgangsregelung. BGBl. I Nr. 12/2020 last modified by BGBl. I Nr. 104/2020. https://www.ris.bka.gv.at/eli/bgbl/i/2020/12/P6/NOR40232039.

[23] Taylor S. Understanding and managing pandemic-related panic buying. J Anxiety Disord. 2021;78:102364. 33517219 10.1016/j.janxdis.2021.102364

[24] Nicola M, Alsafi Z, Sohrabi C, Kerwan A, Al-Jabir A, Iosifidis C, et al. The socio-economic implications of the coronavirus pandemic (COVID-19): a review. Int J Surg. 2020;78:185–93. 32305533 10.1016/j.ijsu.2020.04.018 PMC7162753

[25] Deolmi M, Pisani F. Psychological and psychiatric impact of COVID-19 pandemic among children and adolescents. Acta Biomed. 2020;91:e2020149. 33525229 10.23750/abm.v91i4.10870 PMC7927507

[26] Jones EAK, Mitra AK, Bhuiyan AR. Impact of COVID-19 on mental health in adolescents: a systematic review. Int J Environ Res Public Health. 2021;18. 33802278 10.3390/ijerph18052470 PMC7967607

[27] Nearchou F, Flinn C, Niland R, Subramaniam SS, Hennessy E. Exploring the impact of COVID-19 on mental health outcomes in children and adolescents: a systematic review. Int J Environ Res Public Health. 2020;17. 10.3390/ijerph17228479 PMC7698263 33207689

[28] Statistik Austria. Jahresdurchschnittsbevölkerung 1870–2020. 2021. https://www.statistik.at/web_de/statistiken/menschen_und_gesellschaft/bevoelkerung/bevoelkerungsstand_und_veraenderung/bevoelkerung_im_jahresdurchschnitt/index.html.

