# Supplementary material for: Prescriptions of psychopharmacologic drugs in Austria in 2019 and 2020 – Implications of the COVID-19 pandemic
Source: Eur Psychiatry. 2022 Oct 25;65(1):e73. doi: 10.1192/j.eurpsy.2022.2328 (PMC9677449; doi:10.1192/j.eurpsy.2022.2328)
Supplement: Supplementary file 1 [file S0924933822023288sup001.docx]

**Online Figure 1.** Number of defined daily doses (DDD) of prescribed psychopharmacologic drugs in Austria in 2020 by major drug classes and week of year. The time periods of lockdown 1 (2020-03-16 to 2020-05-01, weeks 12-18) and lockdown 2 (2020-11-17 to 2020-12-06, weeks 47-49) are shaded light-gray.
